# The Concept of an Ideal Antibiotic: Implications for Drug Design

**DOI:** 10.3390/molecules24050892

**Published:** 2019-03-03

**Authors:** Márió Gajdács

**Affiliations:** Department of Pharmacodynamics and Biopharmacy, Faculty of Pharmacy, University of Szeged, 6720 Szeged, Hungary; gajdacs.mario@pharm.u-szeged.hu; Tel.: +36-62-341-330

**Keywords:** antibiotic, multidrug-resistance, drug discovery, ESKAPE, prodrug, persisters, biofilm, metronidazole, *Mycobacterium*

## Abstract

The emergence and spread of antibiotic-resistant pathogens is a major public health issue, which requires global action of an intersectoral nature. Multidrug-resistant (MDR) pathogens—especially “ESKAPE” bacteria—can withstand lethal doses of antibiotics with various chemical structures and mechanisms of action. Pharmaceutical companies are increasingly turning away from participating in the development of new antibiotics, due to the regulatory environment and the financial risks. There is an urgent need for innovation in antibiotic research, as classical discovery platforms (e.g., mining soil *Streptomycetes*) are no longer viable options. In addition to discovery platforms, a concept of an ideal antibiotic should be postulated, to act as a blueprint for future drugs, and to aid researchers, pharmaceutical companies, and relevant stakeholders in selecting lead compounds. Based on 150 references, the aim of this review is to summarize current advances regarding the challenges of antibiotic drug discovery and the specific attributes of an ideal antibacterial drug (a prodrug or generally reactive compound with no specific target, broad-spectrum antibacterial activity, adequate penetration through the Gram-negative cell wall, activity in biofilms and in hard-to-treat infections, accumulation in macrophages, availability for oral administration, and for use in sensitive patient groups).

## 1. Introduction

The discovery and clinical use of antibiotics may be considered to be one the greatest achievements in the history of medicine [1]. The emergence and spread of antibiotic-resistant pathogens is a major public health issue, which requires global action of an intersectoral nature, involving patients and healthcare professionals (prudent use and prescribing [2,3,4,5]), researchers and pharmaceutical companies (development of novel drug candidates, clinical trials [6]) and relevant government stakeholders (government action, financial support [7]) alike. A wide arsenal of bacterial resistance mechanisms has been described, aiding pathogens in evading the lethal effects of these drugs, the most important mechanisms being enzymatic degradation (e.g., β-lactamases, aminoglycoside-degrading enzymes), target alteration (e.g., penicillin-binding proteins, bacterial topoisomerases), decreased uptake (porin-deficient mutants) and overexpression of efflux pump proteins (e.g., AcrAB-TolC in *Enterobacteriaceae*) [8,9]. Multidrug resistant (MDR) bacteria can withstand potentially lethal doses of antibiotics with various chemical structures and mechanisms of action [10,11]. The European Society for Clinical Microbiology and Infectious Diseases (ESCMID) conceived a practical definition for multidrug resistance, where a pathogen is classified as MDR, if they show resistance against three or more antibiotic classes *in vitro* [12,13]. Major public health authorities, such as the World Health Organization (WHO), the European Center for Disease Prevention and Control (ECDC), and the Centers for Disease Control and Prevention in the US (CDC) have all published reports on the significance and the attributable extra mortality that is associated with MDR pathogens [14,15,16,17]. All of these reports concluded that antibiotic resistance is a global issue that may become the major cause of mortality by 2050 [14]. From the standpoint of antimicrobial research, the so-called “ESKAPE” pathogens (E: *Enterococcus faecium*, S: *Staphylococcus aureus* or recently *Stenotrophomonas maltophilia*, K: *Klebsiella pneumoniae* or recently C: *Clostridioides difficile*, A: *Acinetobacter baumannii*, P: *Pseudomonas aeruginosa*, E: *Enterobacter* spp., or recently *Enterobacteriaceae*) receive the most attention, when it comes to identification and screening of novel compounds [18,19,20,21]. This acronym (which was originally coined by the Infectious Diseases Society of America; IDSA) lists MDR bacteria that are of particular concern for healthcare [22]. In addition, extensively drug-resistant (XDR) and pandrug resistant (PDR) strains of Gram-negative bacteria (predominantly *A. baumannii* and *K. pneumoniae*) leave physicians with very few options that are left for treating their patients [23,24].

In the 21st century, it is becoming obvious that the pace of antibiotic drug discovery cannot keep up with the continuous and detrimental changes in resistance trends [25]. In the “golden age” of antibiotic discovery (1960–1980), there were similar developments in bacterial resistance; however, the emergence of novel antibiotic drugs (most of the antibiotic classes currently available were established by the end of the 1980s) or structurally-modified active derivatives of old drugs were potent enough to tip the scales in our favor [26]. This resulted in a shift in interest towards the treatment of chronic illnesses by pharmaceutical companies and governments, and consequently, the development of new antibacterial drugs has markedly slowed down [27,28]. However, since the introduction of fluoroquinolones (which were developed in an attempt to optimize nalidixic acid) in the 1960s, no broad-spectrum agents have been discovered: linezolid and daptomycin are only relevant for the treatment of life-threatening Gram-positive infections, while polymixins (cyclic polypeptides with pronounced toxicity, that were unattractive drugs at the time of their discovery) were re-introduced to therapy, due to the increasing prevalence of MDR Gram-negative infections [29,30,31,32,33]. Ceftaroline–avibactam (a combination of the anti-MRSA cephalosporin and a novel non-β-lactam β-lactamase inhibitor) is the first new drug formulation in a long time that may possess clinically relevant broad-spectrum antibacterial activity [34,35].

Pharmaceutical companies are increasingly turning away from participating in the development of new antibiotics, with large firms like Novartis, AstraZeneca, Sanofi, Bristol-Myers Squibb, and Allergan dropping their antimicrobial research programmes. There are several economic considerations that may explain this phenomenon [36]. The costs of research and development (R&D) and the organization of clinical trials carries a big financial risk irrespective of the drug candidate, and antibacterial drugs only offer modest returns in investments compared to other classes of drugs (e.g., antihypertensive drugs, cholesterol-lowering medications) [37,38]. Novel antimicrobials are typically only used as last-resort agents in critically ill patients, and the duration of therapy is usually limited. In addition, the rapid development of resistance against the new drugs additionally reduces their time period of clinical usefulness [39]. Although there are initiatives and public–private partnerships, such as the *10 × 20 Initiative* of the US Food and Drug Administration (FDA; aiming to produce 10 new systemic antibiotics by the year 2020) and the *New Drugs 4 Bad Bugs (ND4BB) programme* from the Innovative Medicine Initiative (IMI) of the European Medicines Agency (EMA), antibiotic development is largely in the hands of smaller startup biotechnology companies with specific interest in an antibiotic class or infectious disease [40,41,42]. If the number of novel antibiotic classes in the last 50 years is any indication, there is a very low probability for a biologically active compounds to succeed from the pre-clinical to clinical phase of drug discovery. For this reason, reliable discovery platforms are needed to continuously produce compounds with antibacterial activity that may be lead compounds for further studies [43,44]. In Table 1, the currently defined antibiotic discovery platforms are summarized.

The Waksman-platform has dominated the field of antibiotic discovery for almost 40 years, but after overmining soil bacteria, and the continuous re-discovery of already known compounds, this platform was abandoned by pharmaceutical companies [45,51]. There were high hopes for the introduction of high-throughput screening (HTS) methods and rational drug design (RDD) in antibacterial discovery. HTS includes the isolation of bacterial proteins that are essential for survival, and during an automated process, many compounds can be screened for their binding affinity. RDD involves the analysis of the 3D-structure of the target proteins or protein–ligand interactions and developing compounds to interact with specific protein sites [38,58]. Nevertheless, the use of these methods did not meet expectations, as there are hardly any drugs in current clinical use that are the products of this platform, mainly because most of the promising lead compounds identified through HTS were unable to penetrate the bacterial cell wall (particularly in Gram-negative bacteria) and actually bind their defined targets [38,58]. Emerging approaches such as the development of efflux pump inhibitors (EPIs) and virulence-modulating compounds offer new hope in the treatment of infectious diseases. These novel compounds act through sensitizing drug-resistant strains to conventional antibiotics (by modulation of the activity of overexpressed transport proteins) or through eliminating bacterial virulence factors that are crucial for causing disease in humans [64,66,67]. The issue of bacterial cell-wall penetration may also be bypassed by the use of bacteriophage-derived enzymes [70]. These enzymes, termed endolysins (and their recombinant/engineered alternatives, called artilysins) are in essence, peptidoglycan–hydrolases that disrupt the bacterial cell wall, leading to cell death [71,72]. They have an important role in the life cycle of bacteriophages, ensuring the release of progeny virions from the bacterial host cells [73]. This novel approach is promising, owing to their high degree of host specificity; in addition, they could be used as monotherapy or in combination with already existing antibiotics [70,73]. Still, these compounds are currently relevant only in experimental settings, as none of these have been cleared for clinical use. For further reading on antimicrobial discovery platforms mentioned above (Table 1.), the reader is encouraged to view the excellent publications of Kim and Kealey et al. [38,45,46].

In addition to discovery platforms, a concept of an ideal antibiotic should be postulated, to act as a blueprint for future drugs [74]. The intent of this model is to direct antibacterial discovery and drug design, and to aid researchers, pharmaceutical companies, and relevant stakeholders in selecting promising lead compounds, moving forward in the “maze” of this field. Based on the properties that are set for this theoretical molecule, screening methods may also need to be adjusted and optimized [55]. The aim of this review is to discuss the current advances regarding the attributes of an ideal antibacterial drug.

## 2. The Ideal Antibiotic (Prodrug) Model

The ideal antibiotic should have broad-spectrum bactericidal activity (although the clinical relevance in the difference between bacteriostatic and bactericidal drugs has been questioned by multiple studies [75,76,77]), against bacteria with Gram-positive and Gram-negative cell walls, *Mycoplasma*/*Ureaplasma* ssp. (bacteria with no cell wall [78]) and L-form (cell wall-deficient [79,80,81]) bacteria. Persisters (defined as metabolically inactive bacterial cells that neither grow or die when exposed to bactericidal concentrations of antibiotics) present another important challenge to antimicrobial therapy that has yet to be approached from the standpoint of drug discovery [82]. These dormant cells usually represent a very minor fraction of the population in the exponential growth phase; however, they may represent up to 1% of cells in the stationary phase, during long-term antibiotic therapy and in a biofilm [83]. Therefore, they have been associated with therapeutic failure, recurrence, and chronic infections, as they may continue to replicate after the antibiotic therapy has been discontinued [84]. The production of biofilms is considered a survival strategy to adapt to a hostile living environment. Infections associated with biofilms are an increasingly important issue, especially due to the prevalence of nosocomial infections and the use of indwelling catheters and prostheses [85,86]. The production of biofilms in cystic fibrosis patients is an additional concern, because antibiotics cannot successfully penetrate to affect the planktonic phase of growth in these cells, contributing to the morbidity and mortality of the disease [87]. Some antibiotics (such as rifampin) can penetrate and break up this extracellular polymeric matrix produced by bacteria, which is why they are usually used in combination with other drugs to enhance their efficacy [85,88,89].

The penetration barrier of Gram-negative cell wall is an important obstacle for antimicrobial development [90]. The outer membrane (OM) of Gram-negatives restricts amphipathic drugs from crossing through, while the inner membrane (IM) restricts hydrophilic substances from entering the cell. This essentially creates a very potent barrier, which allows for the penetration of only a select number of antimicrobials [91]. Therefore, penetration rules may also be established, similarly to rules of oral bioavailability (e.g., the Rule of Five, see below). Based on the library of compounds with good penetration through the Gram-negative cell wall, common physico-chemical characteristics could be identified [92]. Small, hydrophobic compounds (such as aminoglycosides and chloramphenicol) can diffuse through the lipid component of the OM, while β-lactam antibiotics predominantly move through porin channels to reach their targets in the periplasmic space [93,94]. The latter carries a risk of resistance development, because porin mutants (prevalent in *Pseudomonas aeruginosa*) usually lose their susceptibilities to these drugs [95,96]. The over-expression of efflux pumps (which is a concern in MDR Gram-negative bacteria) is also a significant mechanism of resistance [97,98]. These transport proteins, due to their wide substrate specificity, can extrude various noxious agents (toxins, bile salts, antiseptics and antibiotics), although their preference towards amphipathic drugs have been described [64,99]. The use of EPIs present as adjuvants is an attractive strategy; still, a compound that is not affected by these pumps would be the most advantageous.

This ideal molecule should be highly reactive, forming an irreversible, covalent bond on multiple, unrelated targets, leading to bacterial cell death [38]. This is important for two reasons: firstly, covalent binding guarantees that the molecule will accumulate inside the bacterial cell and will not be extruded by energy-(ATP-dependent cassette-transporters) or H^+^/Na^+^-gradient-dependent efflux transporters (e.g., major facilitator superfamily transporters); secondly, reacting with multiple targets ensures that drug resistance may not develop through single-step mutations (e.g., quinolone resistance) and target modification (e.g., macrolide-lincosamide-streptogramin [MLS] resistance) [64,100]. An emerging concept is that the molecule should function as a prodrug (or be formulated as such), which has little or no effect on mammalian cells, but that will kill all bacterial cells, including persisters. To attain this, the prodrug molecule should be activated by an enzyme that is specific to and abundant in pathogenic bacteria, resulting in an end-product that is extremely reactive. This is the reason for why the concept of an ideal antibacterial drug is also called the prodrug model [38].

In addition to the interactions of the molecule with the target microorganisms during therapy, these compounds must meet a set of pre-determined set of physico-chemical characteristics that a lead compounds should possess in order to become a drug candidate [101]. Based on data from the United States, more than 80% of drugs in current use are orally administered; therefore this route should be primarily targeted [102,103]. This is especially true for the treatment of infectious diseases, where intravenous (IV) administration should only be used, if it is justified by the medical condition of the patient. By definition, antibiotics with >90% bioavailability (doxycycline, minocycline, clindamycin, metronidazole, trimethoprim-sulfamethoxazole, linezolid, tedizolid, and rifampin) are candidates for IV-to-PO interchange (exceptions are ciprofloxacin (~70% bioavailability) and azithromycin (~40% bioavailability), as they still manage to achieve the therapeutic levels taken orally) [104]. Such IV-to-PO switches (i.e., sequential antibiotic therapy) are further encouraged in the era of antimicrobial stewardship. In order to attain good oral bioavailability, Lipinsky’s Rule of Five (RO5) is generally used as a preliminary indicator of drug-likeness during pre-clinical studies [105]. These rules (*a. ≤ 5 hydrogen bond donors, b. ≤ 5 hydrogen bond acceptors, c. molecular mass <500 Da, d. octanol-water partition coefficient (clogP) < 5) assumed that the most commercially successful, orally administered* molecules are relatively small and moderately lipophilic [106,107]. However, this may create a very narrow window of compounds that are eligible to penetrate Gram-negatives and that are orally bioavailable. In addition, screening based on these rules may exclude potential leads, because they do not consider the differential properties required to penetrate prokaryotes [88]. To further ease the formulation of oral drugs, the compound should be a Class I molecule in the Biopharmaceutical Classification System (BCS) [108].

Tissue penetration of the molecule should be adequate to attain therapeutic concentrations in all parts of the body, including peripheral areas, and in infected sites that are hard-to-reach and that have specific physico-chemical characteristics (e.g., abscesses, central nervous system, bone tissue) [109,110]. Additionally, the accumulation of antimicrobial drugs in macrophages and non-professional phagocytes (i.e., in the phagolysosome of these cells) are also relevant in the elimination of obligate (*Chlamydia spp., Rickettsia spp., Coxiella spp., Mycobacterium tuberculosis and leprae*) and facultative (*Listeria monocytogenes*, *Legionella pneumophila*, *Brucella abortus*, *Bartonella henselae*, *Francisella tularensis*, *Salmonella enterica*, and other *Mycobacterium* species) intracellular bacteria [111,112,113]. A few antibiotic groups (e.g., macrolides) are known for their effective intracellular accumulation, and some new agents that are receiving marketing authorization (such as delafloxacin) also possess this attribute [114,115,116,117].

Compared to other drugs, antibiotics are effective in concentrations that are two to four magnitudes higher than other molecules affecting distinct molecular targets in the human body [104]. This carries a risk of inherent toxicity, excluding most of the potential compounds from being potential leads. Therefore, it is imperative that the abovementioned prodrug form of the antibiotic should have no affinity to bind to eukaryotic targets before entering the bacterial cell [45]. Another emerging aspect of antimicrobial pharmacotherapy is the treatment of infections during pregnancy, lactation, and in childhood. Therapy in these patient groups in practically limited to β-lactam antibiotics, due to the teratogenic and adverse events described in other antibacterial drugs [118,119,120]. Therefore, an additional aim should be to produce drugs that are available for use in these vulnerable patient groups. Some regulatory agencies provide additional periods for patent exclusivity (pediatric exclusivity), to incentivize drug development in pediatric indications [121]. Drug–drug interactions are significant hindering factors in the efficacy of drugs, predominantly due to their inducing or inhibiting effect on various cytochrome P450 enzymes (predominantly the CYP3A4, CYP2C9 and CYP2D6 isoenzymes), affecting therapeutic response by modulating the degradation of other medicinal drugs [122,123]. An ideal antibiotic should be metabolized without affecting liver enzymes and it should be eliminated from the body unaltered (e.g., in the urine).

## 3. Prodrug Antibiotics in Clinical Use

**Figure 1:**
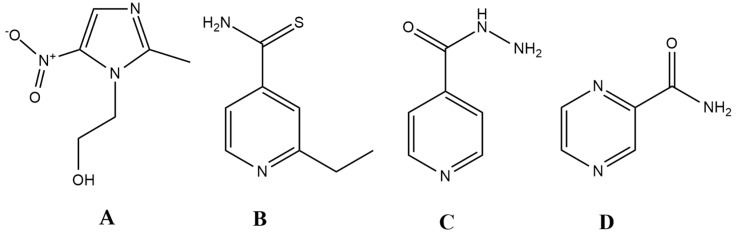
Antibiotics that closely resemble the properties set up by the ideal antibiotic (prodrug) model. (**A**): metronidazole; (**B**) ethionamide (ETH); (**C**) isoniazid (INH); D: pyrazinamide (PYR).

The question arises as to whether the ideal antibiotic can only be a theoretical concept or is it realistic to identify and design such molecules. Surprisingly, there are a few drugs in current clinical use that have similar characteristics to this model, namely *ethionamide*, *isoniazid*, *pyrazinamide* and the *metronidazole-like* drugs (Figure 1.). *Metronidazole* is a broad-spectrum, bactericidal antibiotic, which is available in both oral and intravenous formulation [104]. In addition, it is relevant in other fields of infectious diseases, owing to its potent antiprotozoal activity (against *Giardia lamblia*, *Trichomonas vaginalis*, *Entamoeba* sp.). This drug belongs to the 5-nitroimidazole group drugs, together with its derivatives, *tinidazole*, *ornidazole*, *ronidazole* and *secnidazole*. Moreover, these compounds can be considered as the primary lead compounds for *nitazoxanide* (and its active metabolite *tizoxanide*), which are broad-spectrum antiparasitic agents [124]. Metronidazole is an important drug for the treatment of *Helicobacter pylori,* and it represents the gold standard in drug therapy for anaerobic infections [125,126,127,128]. Apart from some Gram-positive anaerobes (*Mobiluncus curtisii* and the genera *Actinomyces*, *Bifidobacterium*, *Lactobacillus* and *Propionibacterium*) having intrinsic non-susceptibility, the resistance to this drug is <1% worldwide [129,130,131]. Metronidazoles act as a prodrug, and it must be reduced by specific enzymes (namely nitro-reductases and redox-active enzymes, such as pyruvate:ferredoxin/pyruvate:flavodoxin oxidoreductase and hydrogenase), during which an electron is transferred to the nitro group of the drug [132]. The resulting nitroso-residues are non-specific, highly reactive, and have a short half-life, damaging the bacterial cell membrane, DNA (inducing strand breakage and destabilization of the helix structure), and proteins. Unfortunately, these enzymes are only expressed in pathogens that live under microaerophilic and/or anaerobic conditions. In addition, chemical reoxidation may also occur if molecular oxygen is present, converting the compound back to its inactive form [130]. Metronidazole is available in both oral and intravenous formulations; its bioavailability is almost 100%, and it has excellent tissue distribution. 

Ethionamide (ETH), isoniazid (INH), and pyrazinamide (PYR) are all drugs that are relevant for the treatment of the *Mycobacterium tuberculosis* complex. Generally, INH and PYR are part of the first-line treatment regimen for TB, together with rifampicin and ethambutol, while ETH (and its therapeutic alternative *prothionamide*) is usually considered as a second-line drug, useful in drug-resistant TB [133,134]. All three drugs are bactericidal, and they can penetrate well into macrophages, which is an important aspect of treating the disease, as mycobacteria use macrophages to hide from the immune system [104,134]. They also turn into active derivatives after interaction with a *Mycobacterium*-specific enzyme: ETH requires activation by EthA (a flavin mono-oxygenase) and INH is activated by KatG (a catalase-peroxidase), while PYR is converted to its active form by the PZase/nicotinamidase, encoded by the pncA gene [135,136,137,138,139]. In the case of INH and ETH, following enzymatic activation, these metabolites form an adduct with nicotinamide adenine dinucleotide (NAD+), resulting in ethionamide-S-oxide-NAD and isonicotinic-acyl-NAD adducts; these metabolites are responsible for the antitubercular activity of the parent compounds [136,137,138,139]. In the case of PYR, activity against persisters has also been described, a property that is attributed to its active form, pyrazinoic acid (POA), which retains activity in cells with low metabolic activity [135]. Nevertheless, specific targets for all three drugs (namely, trans-2-enoyl-acyl carrier proteins (ACPs) for INH, ribosomal protein S1 (RpsA), and/or membrane destabilization for PYR and arabinozyl-transferase for ETH) have been identified, while the ideal antibiotic should hit multiple targets in a non-selective fashion [136,137,138,139]. This points to the notion that these drugs may not be as reactive as metronidazole.

It seems no surprise that all the above-mentioned drugs (Figure 1) are listed in the Essential Medicines List of the WHO, indicating their importance and the need for universal access [140]. This is further highlighted by the fact that INH and PYR represent half of the current first-line drugs for TB [141]. It is worth mentioning that all of the compounds corresponding to the prodrug rules are relatively small molecules (with molecular weights ranging between 123–171 g/mol); they have been discovered before the advent of HTS technologies and rational drug design, and no such compounds have been described since. This is especially odd, as the number of new compounds (i.e., the chemical space) is many magnitudes larger than half a century ago [45]. Through optimizing our discovery and screening platforms, the possibilities of finding compounds that—in classical pharmacological terms—have no specific targets is very limited (as most pre-clinical screening assays usually measure binding affinity). Redox-active compounds and drugs acting primarily on the cell membrane are groups of molecules that would definitely go unnoticed in these experiments. Based on the current screening criteria, the first sulfonamide drug (*Prontosil*) would have been excluded, as the active compound *sulfanilamide* becomes available only after in vivo metabolism [49]. Similarly, metronidazole (as it is a generally reactive compound with no specific target) and the polymyxins (possessing a detergent-like mechanism of action) would be considered undesirable leads. Nonetheless, the importance of these drugs should not be underestimated. In fact, some studies reported that all antibiotics may act via a unified mechanism of action, through the generation of reactive oxygen species (ROS) and direct cellular damage; however, there have been conflicting reports in this field of research [142,143,144,145,146].

## 4. Concluding Remarks

The growing number of antibiotic-resistant pathogens is increasingly threatening the efficacy of healthcare institutions worldwide. Antibiotic discovery needs to be re-energized, to rival the threat of the post-antibiotic era [25]. The attributes of the ideal antibiotic—summarized in Table 2—may be divided into pathogen-specific and drug-specific properties; however, this classification is somewhat arbitrary, as there is notable interplay between fulfilling both groups of characteristics. Furthermore, some important aspects of drug development and medicinal chemistry (yields of potential synthetic pathways, economic considerations of production, stability of the compound in various formulations) were not discussed in this review.

Realistically, producing a molecule that possesses all the listed properties above is very unlikely; therefore, the usefulness of this model is to aim towards specific features from the list, based on the pathogen, site of infection, administration route, and the targeted patient population during drug development. As a matter of fact, the best possible scenario would be to modify and/or functionalize existing antibiotics to attain more of the mentioned properties. Although there are some alternative approaches in development for combating infectious diseases (e.g., antibodies, probiotics, vaccine development, phage therapy, small-molecule adjuvants affecting immune cells), it is unreasonable to believe that they will replace antibiotics anytime soon [147]. Therefore, the main foci of our scientific advancements should be to preserve the drugs that we currently have (through the development of rapid and sensitive diagnostic tools to ensure their prudent use, and antibiotic stewardship practices [148,149,150]), in addition to facilitating the development of new antibacterial drugs.

## Figures and Tables

**Table 1:** Overview of various discovery platforms for antibacterial drugs [38,45,46].

Platform	Brief Description of Pros and Cons	Compounds in Clinical Practice (Examples)
Domagk-platform/In situ screening-platform	Screening the efficacy of antimicrobial compounds at the site of infection (with the use of infection models; e.g., in an in situ mouse model or in a *Caernorhabditis elegans* worm model [47,48]) Detects prodrug compounds that would be missed by high-throughput screening and validation approaches [49] Ethical considerations (related to the use of animal models)	Sulfonamides (sulfamidochrysoidine)
Waksmann-platform/Natural products-platform	Screening for secondary metabolites in soil microorganisms (*Streptomycetes)* with antibacterial activity [50] Main discovery platform in the golden era of antibiotic discovery [51] Background of known compounds during screening presents a major issue [45] Experiments are ongoing with the activation of “silent operons” in microorganisms [52] Focusing on uncultured microorganisms (representing 99% of total microbial diversity) and compound de-replication (using mass spectrometry and nuclear magnetic resonance (NMR)) are promising approaches [53] Screening for antibacterial compounds from plant and marine origins represents an untapped resource of potential drugs [54,55]	Penicillin (First antibiotic discovered) Streptomycin (First drug active against tuberculosis (TB)) Daptomycin (MDR Gram-positives) Fidaxomicin (*Clostridioides difficile*)
Species-selective platform	Screening against a specific bug, resulting in compounds that act selectively against that pathogen [56] Requires a target that is innate and specific to microorganism Lower probability of toxicity in the human host New compounds will not affect commensals in the gut [57]	Bedaquiline F_1_F_0_-ATPase-inhibitor in *Mycobacterium tuberculosis complex* Ethambutol Arabinosyl-transferase-inhibitor in *Mycobacterium tuberculosis complex*
High-throughput screening (HTS) Combinatorial chemistry (CC) Rational drug design (RDD)	Screening of public/commercially available libraries of compounds against bacterial strains and/or defined prokaryotic targets (ligand–target binding assay, specificity tests) [58]	Oxazolidinones Inhibitors of protein synthesis by interfering with the ribosomal 50S subunit
Antimicrobial peptides (AMPs)	Use of small-sized, positively charged, amphipathic molecules synthesized by plants, animals or other bacteria [59] They play an important role in innate immunity in humans (e.g., defensins) [60] Structurally, they may be α-helices, β-sheets or extended coils, all with different mechanisms of action [61] Toxicity in humans in higher concentrations [61] Difficulties in formulation [62]	No AMP has been approved yet for clinical use
Resistance reversing compounds	Compounds affecting a defined mechanism of bacterial resistance, e.g., antibiotic-degrading enzymes, efflux pumps [3] Strains that are resistant to specific antibiotics may be sensitized, maintaining the efficacy of current drug pool [63,64,65] The clinical relevance of efflux pump inhibitors (EPIs) is hard to determine	Beta-lactamase inhibitors (clavulanic acid, sulbactam, tazobactam, avibactam etc.) No EPI has been approved yet for clinical use
Virulence modulation	Compounds targeting expression and/or activity of bacterial virulence factors (capsule, toxins, fimbriae, biofilm) essential in their pathogenesis [66,67] Various small-molecule compounds (e.g., quorum sensing-inhibitors) and monoclonal antibodies have been described [68,69] Selective pressure to develop resistance is not present [68] The clinical relevance of virulence modulators is hard to determine	No virulence modulator has been approved yet for clinical use

**Table 2:** Summary of the properties of the ideal antibiotic.

Drug-Specific	Pathogen-Specific
Available for oral administration	Broad-spectrum bactericidal activity (including Gram-positive and Gram-negative bacteria, *Mycoplasma*/*Ureaplasma* ssp. and intracellular pathogens)
Acts as a prodrug	Antibacterial activity against persisters and pathogens in biofilms
Class I in the Biopharmaceutical Classification System	Activity at very low (nanomolar) concentrations
Accumulation in macrophages	Useful in hard-to-reach infected sites, e.g., abscesses, central nervous system (CNS), bone tissue
No teratogenic effects (safe in pregnancy, lactation and childhood)	Acts on multiple, unrelated, essential bacterial targets
No drug–drug interactions	Forms irreversible covalent bonds inside bacterial cells (ruling out drug efflux)
The drug is excreted from the body unchanged	

## Abbreviations

ACP	acyl carrier protein
AMP	antimicrobial peptide
BCS	Biopharmaceutical Classification System
CC	combinatorial chemistry
CDC	Centers for Disease Control and Prevention
CNS	central nervous system
ECDC	European Center for Disease Prevention and Control
EMA	European Medicines Agency
EPI:	efflux pump inhibitor
ETH	ethionamide
FDA	Food and Drug Administration
HTS	high-throughput screening
IDSA	Infectious Diseases Society of America
IM	inner membrane
IMI	Innovative Medicines Initiative
INH	isoniazid
IV	intravenous therapy
MDR	multidrug resistant
MRSA	methicillin-resistant *Staphylococcus aureus*
NAD	nicotinamide adenine dinucleotide
ND4BB	New Drugs 4 Bad Bugs
OM	outer membrane
PO	oral therapy
POA	pyrazinoic acid
PYR	pyrazinamide
PDR	pandrug resistant
RDD	rational drug design
ROS	reactive oxygen species
R&D	research and development
RO5	Lipinsky’s Rule of Five
TB	tuberculosis
XDR	extensively drug resistant
WHO	World Health Organization
